# Dimensionality of Early Adversity and Associated Behavioral and Emotional Symptoms: Data from a Sample of Japanese Institutionalized Children and Adolescents

**DOI:** 10.1007/s10578-018-0850-4

**Published:** 2018-10-27

**Authors:** Yuning Zhang, Charlotte C. A. M. Cecil, Edward D. Barker, Shigeyuki Mori, Jennifer Y. F. Lau

**Affiliations:** 1 Department of Psychology, Institute of Psychiatry, Psychology & Neuroscience, King’s College London, 16 De Crespigny park, London, SE5 8AF UK; 2 Department of Child and Adolescent Psychiatry, Erasmus Medical Centre, Rotterdam, Netherlands; 3 Konan Institute of Human Sciences, Konan University, 8-9-1 Okamoto, Higashinadaku, Kobe, Japan

**Keywords:** Childhood adversity, Dimension, Symptomatology, Japan

## Abstract

Recent approaches have begun to identify common variance across co-occurring childhood adversities (CAs) and their associations with symptoms of psychopathology. However, few studies have investigated these questions in high-risk samples, and in different cultural contexts. This study examined common variance amongst 18 types of CAs and associated symptomatology in 457 children and adolescents living in 24 residential homes in Japan. Principal component analysis identified four significant components that explained 35.1% of the variance: parental abuse, parental psychosocial risks, parental absence, and parental neglect. Path analysis revealed general as well as differential associations with negative outcomes: parental abuse, parental neglect, and parental psychosocial risks significantly associated with conduct problems, whereas parental abuse uniquely associated with peer problems, and parental neglect with hyperactivity/inattention. As well as confirming prior knowledge, these findings also extended understanding of these associations to a new cultural context. Future studies should take into account the multidimensional nature when assessing CAs.

**Electronic supplementary material:**

The online version of this article (10.1007/s10578-018-0850-4) contains supplementary material, which is available to authorized users.

## Introduction

Childhood adversity (CA) is an umbrella term for environmental circumstances or events that deviate from the “expected” normative/typical environment such as childhood maltreatment and poverty [1–3]. Collectively, these events robustly predict later internalizing and externalising outcomes in youth. However, rather than to consider the individual and additive effects of these events on later psychopathology, researchers have recently begun to identify common variance amongst CAs on outcomes. Notably, events that reflect physical or emotional *threat* to an individual have been distinguished from events that reflect *deprivation* (e.g. through neglect and poverty) [4, 5] on the basis of their differential associations with cognitive functions in community samples of adults as well as adolescents [4, 6]. Whether threat and deprivation exhaustively reflect adverse childhood events and also show differential associations with behavioural and emotional outcomes in those at higher risk for psychopathology remain outstanding questions. The present study aims to address these questions by assessing the dimensionality of a broad spectrum of early experiences, and their associations with externalising and internalizing symptomatology, amongst a high-risk child and adolescent sample in Japan. A secondary aim is to also explore the cross-cultural validity of these existing findings to enhance their generalizability more globally.

A longstanding challenge to studying the effects of CAs on later psychopathology has been how to capture their combined versus unique effects on different developmental outcomes. While earlier studies tended to ignore the interplay between CAs by summing their effects on outcomes [7–9], more recent approaches have applied latent trait models (e.g., factor analysis, latent class analysis, principal component analysis) to assess common and distinct variance between CAs [4, 5, 10]. Such approaches have found that within parental maltreatment, abusive events and neglect seem to cluster separately [4, 5], providing support for a recently proposed theoretical framework [11], which suggests that *threat* (representing a series of experiences that are of threat to one’s physical integrity) should be distinct from *deprivation* (representing the absence of expected cognitive and social input).

Although this emerging approach has informed the multidimensional nature of early adverse experiences, a number of outstanding questions remain. First, although threat and deprivation reflect key dimensions within parental maltreatment, they may not capture all aspects of family malfunctioning. Within this broader category, other CAs, for example, parental criminality, parental substance use and parental violence, seem to co-occur, and may be distinct from parental abuse and/or neglect [4, 5]. Second, it may be that there are distinct forms of deprivation such that events representing circumstantial deprivation (e.g., parental loss) are distinct from physical and/or emotional neglect. Identifying other distinct dimensions of CA and further sub-dividing existing dimensions could be better informed by assessing a wide range of adversities, such as those present in a high-risk sample who experience more extreme forms and more combinations of CAs [12, 13]. So far, as most existing studies have drawn on general population samples, co-occurrence between CAs may be limited due to floor effects of total number of CAs experienced by participants.

A second outstanding question is that most of this research of the specificity of associations between dimensions of CAs and outcomes has largely focused on finding specificity within neurocognitive functional domains, such as fear processing [14], and social cognitive abilities [4]. Little is known about whether these distinct dimensions of adverse experiences translate to psychopathology. Studies assessing broad categories of CAs (e.g., childhood maltreatment, extreme forms of deprivation such as early institutionalization) have reported both common [15–18] and specific [19, 20] associations with internalising and externalising problems. This mixed picture could reflect difficulties in disentangling individual dimensions of adversity within broad CA categories. While less research had focused on internalizing difficulties, studies using natural experimental designs have provided valuable insights on specific associations with externalising problems. Data from the English Romanian Adoptee Study, for example, showed that patterns of disinhibited attachment, impaired cognitive abilities, hyperactivity, and quasi-autistic behaviours reflect a constellation of deprivation-specific psychological consequences [21]. Other studies have also reported a high prevalence of hyperactivity and disinhibited social engagement amongst children who have experienced severe deprivation from institutional rearing [21–23]. Conduct problems, on the other hand, often arise following the experience of abuse [16] or interpersonal violence [24]. These studies, while insightful, are still limited by the co-occurrence of adversities, e.g., institutional deprivation is likely to co-occur with peer victimization even when parental abuse is absent [25, 26]. These co-occurring adversities could confound findings. Given these limitations, a latent trait model can potentially clarify these mixed results by identifying common variance across CAs. For example, Copeland and colleagues reported that although both suffered from poverty, children exposed to single parenthood and/or parental crime exhibited elevated disruptive behaviour, whereas children with parental poor education (at least one parent left school before 11th grade) were at higher risk of emotional disorders [10].

It is also worth noting that there are comparatively few studies assessing the dimensional nature and their impact conducted in countries such as those from the Far East compared to the rich evidence-base from Europe and North America. Nonetheless, the limited evidence suggests country-based differences in responses to early-life adversities. For example, compared to rates of adversities reported from general population based adult samples in the USA [27], Japan reported fewer adversities and moreover, the association with psychopathology was less generalized and did not persist beyond adolescence [28]. In contrast, countries such as Mexico [1] and China [29] showed more comparable data to the USA. Extending such evidence of how these differential pathways translate into symptom types can inform the generalizability of existing findings to a global context.

The present study aimed to address these gaps, by assessing the key dimensions underlying multiple CAs, as well as their association with symptomatology within a high-risk sample. Using data from an institutionalized children and adolescent sample from Japan, we tested two hypotheses. First, we expected that using Principle Component Analysis, the threat-deprivation theme would emerge in our sample together with other potential components of parental malfunctioning, such as circumstantial deprivation. Second, these dimensions were predicted to hold distinctive associations with symptomatology: after controlling for covariates. More particularly, we expected that threat would uniquely associate with both externalising and internalizing symptomatology, and deprivation with externalising symptoms. We investigated these questions in a sample that included adolescents, given that this developmental period is relatively under-studied in terms of the impact of CAs on psychological functioning. Yet, studying the impact of CAs on psychopathology at this juncture is crucial given that many persistent psychiatric problems first onset during adolescence [30, 31], and thus could be a sensitive peirod for intervention [32]. Notably, a secondary aim of the study was to extend the cross-cultural validity of the CA research to Eastern cultures.

## Method

### Study Sample and Sampling Procedure

The study sample comprised of 457 children and young people (44.9% girls) aged between 8 years 0 months and 15 years 3 months (*M* = 11.7 years, *SD* = 1.93 years) from the Japan Jidoyogoshisetsu Study (JJS). All participants were living in institutions in Japan, and the total time spent in care ranged between 2 weeks and 14 years 3 months (*M* = 6.62 years, *SD* = 3.94 years). Age of being removed from biological family varied from immediately after birth to 13 years 4 months (*M* = 5.11 years, *SD* = 3.79 years) (see Table 1 for participant demographics).

**Table 1:** Variable descriptive and associations between covariates and Strength and Difficulties Questionnaire scores

	Mean (SD)^c^ or % (n)	SDQ			
		HI	CP	ES	PP
Covariates					
Age	11.7 (1.93)	− .069	− .024	.014	− .072
Gender (female)^a^	44.9% (n = 205)	.164**	.102*	− .143**	− .006
Total time spent in care	6.67 (3.95)	.139**	.161**	− .008	− .085
Psychological and behavioural symptoms—SDQ^b^					
Hyperactivity/inattention (HI)	5.06 (2.76)	–	–	–	–
Conduct problems (CP)	3.57 (2.49)	–	–	–	–
Emotional symptoms (ES)	2.83 (2.25)	–	–	–	–
Peer problems (PP)	3.61 (2.23)	–	–	–	–

Bivariate correlations significant at: *p < .05; **p < .01; ***p < .001

^a^ Gender (female = 0, male = 1)

^b^ Strength and Difficulties Questionnaire

^c^ Standard deviation

Institutions from four prefectures in east and west Japan (Hyogo, Tokyo, Aichi and Fukushima) were contacted and invited to take part in a study in 2010, aiming to examine the relationship between the institutional rearing environment and children’s psychological wellbeing in Japan. Eighteen institution directors from Hyogo (out of 19 contacted) and six from Tokyo (out of 6 contacted) responded positively to our invitation, whereas one from Hyogo and two from Aichi (out of 2 contacted) declined to participate. Although one institution from Fukushima also agreed to participate, the 2011 Tsunami led to our exclusion of the CWI from data collection. Of 1295 children and adolescents available from the 24 institutions, 592 met the following three inclusion criterias: (1) aged 8–15 years old (pre-adolescence/adolescence), (2) had been in current care for at least 2 weeks, and (3) were not undergoing legal proceedings concerning their placement. Although all participants in the sample would have undergone legal care proceedings in order for their placement to be legally finalised, we excluded those currently experiencing these procedures because of the uncertainty and temporal instability of an ongoing proceeding which could influence the participant’s emotional state and behaviour, confounding the effects of pre-institutionalisation CAs.

Overall, 457 (77.2%) of the 592 eligible young people were available on the day of data collection and agreed to participate. Reasons for unavailability included: (1) absence due to school activities, (2) absence due to biological family visitation, and (3) feeling unwell. Notably, due to ethical restrictions from the local ethics board and the Hyogo Child Welfare Committee, information on youth who did not participate was not made available for researchers. Thus, it was difficult to compare the demographic characteristics between those who did and did not participate. Informed consents were obtained from the institution directors and the key caregivers of target children/adolescents, while assents were sought from participants themselves after an explanation of the study and the opportunity to ask questions was given. Informant reports were completed by children’s key caregivers (*N* = 213) in institutional care, that is, the caregiving staff responsible for the child’s day-to-day activities in institutions and schools, and who act to liaise between the child and social workers and family members (if applicable). Amongst them, 92 caregivers reported on one child only, 66 caregivers reported on two children, and 55 reported on three or more children at the same time. Key caregivers typically receive University-level qualifications and professional training qualifications in child-care, as shown in this study where 69.1% had completed 4-year university-level qualifications or higher, and 90.5% had at least 1 government recognised professional qualification related to the care or education of children. Through their training, they are familiarized with the definitions of CAs and children’s internalizing and externalising symptoms; thus it was thought appropriate to invite them to report on these aspects of the children’s referral history and current functioning. Ethical approval for the study was sought from Konan University in 2011. All procedures performed in the study were in accordance with the ethical standards of Konan university ethical committee and with the 1964 Helsinki declaration and its later amendments.

### Measures

#### Child Characteristics and Placement-Related Background

Key caregivers provided information on participants’ (1) gender, (2) age, and (3) age of removal from biological family, from which total time spent in care was calculated by subtracting age at testing.

#### Childhood Adversity

Due to the unique challenges associated with assessing CAs in a group of high-risk state-protected children and adolescents, we relied on caregivers to complete three checklists: *Referral Reason Checklist* which is an official document containing the primary reason an individual was placed in care; *Family Background Checklist* and *Maltreatment Checklist*, which were both designed by the project to capture children’s adverse experiences that were (1) not noted as the primary referral reason in the referral reason checklist, but noted in the case record, and (2) not picked up and documented by case workers at the time of referral, but became clear in daily interactions between key caregivers and children when they arrived at the CWIs. Due to ethical concerns and safeguarding reasons around the children’s well-being, we were unable to seek permission to interview participants directly about their adverse experiences prior to care placement, nor were we permitted to retrieve information directly from participants’ official case records. Instead key caregivers could access official case records kept at each CWI and, as information providers, were fully briefed on how to complete each checklist. We did not capture information around the intensity and duration of adverse experiences in our data partly because case records vary in the level of detail provided, both across reporters and institutions. Hence to keep this standardised across all participants, we relied on dichotomous ratings (0 = no, 1 = yes) of presence or absence. Of note, dichotomous ratings are preferred for the following reasons: (1) rater variability can be reduced by dichotomous ratings as opposed to dimensional ratings; (2) having these items being rated continuously would require introducing variables such as frequency or severity, which could not be consistently interpreted across participants due to variable amount of details contained in the case records; (3) dichotomous ratings are more straightforward to administer, and require less effort from informants, hence it was most practical for minimizing task demands on the informants.

*Referral Reason Checklist* is an official document that contains an exhaustive list of adversities reported to child-protection services. It is used across all Japanese child protection services as the primary reason for a child’s referral to care proceeding. There are 15 items included: (1) parental death; (2) missing parent(s); (3) parental divorce; (4) parental hospitalization; (5) non-specific unpreferable rearing conditions; (6) poverty; (7) parental imprisonment; (8) homelessness; (9) abandonment at infancy; (10) child’s own developmental problems; (11) physical abuse; (12) emotional abuse; (13) sexual abuse; (14) physical neglect; (15) other. For the definition of each item, please refer to Table 2.

**Table 2:** Definition of items included in the Referral Reason Checklist

Item	Definition
1. Parental death	One or both parents passed away
2. Missing parent(s)	One or both parents has or have abandoned the family without any prior warning/signs and is/are currently uncontactable or unlocatable
3. Parental divorce	Parents divorced, as well as any family conflicts resulted from it
4. Parental hospitalization	One or both parent(s) being hospitalized for physical and/or mental health problems for more than half a year
5. Non-specific unpreferable rearing conditions	To differentiate from absolute poverty, this includes situations such as deterioration in the living conditions of the child, the parent(s) being migrant workers, often leaving the child home alone or with other adults, child not being taken care of due to parental physical or occupational injury, disabilities, or illness, or the family having too many children
6. Poverty	Financial hardship, including parental unemployment or unstable employment, low income compared to the national standard, high rates of family debt
7. Parental imprisonment	One or both parents fail to care for the child due to time spent in prison, regardless of the length of stay
8. Homelessness	The child or family do not have a fixed residence and live in outdoor/public spaces such as parks and streets
9. Abandonment at infancy	Due to various reasons, the child is left abandoned in places such as hospitals, streets, and child welfare institutions, before turning 3 years old
10. Child’s own developmental problems	The child is taken to state care facilities due to developmental difficulties
11. Physical abuse	Includes parental behaviour that involve punching, kicking, throwing, violently shaking, burning, drowning a child, squeezing their neck, restraining them with a rope
12. Emotional abuse	Child being verbally threatened, ignored, discriminated against siblings by parents, or parents being violent toward other family members in front of the child
13. Sexual abuse	Parents, caregivers and other adults exhibiting sexual behaviour towards the child, or showing sexual acts, touching or touching genitals, making them subjects of pornography
14. Physical neglect	The child has been keep/locked in the house, given no meals, left dirty without a cleaning routine, left alone in the car for a long period of time, failed to be taken to the hospital when needed, failed to be given the opportunity of education
15. Other	Anything not mentioned in the 14 items

*Family Background Checklist* assesses three major family risks: recipient of governmental financial aid, parental mental illness history (any mental illnesses, e.g., schizophrenia, depression, alcohol abuse), and parental criminality history. For each risk, caregivers were asked to rate yes (1) or no (0) on whether such an event had occurred to either the biological mother or father of the participant, and the same was done for another nominated member of the family that the participant was known to live with e.g. grandparents. Regardless of the relationship, if such an event existed within the family the item was coded as 1, and coded 0 if no family members had such record. If such circumstances were unknown, it was coded as missing.

*Maltreatment Checklist* required caregivers to report on the existence of four types of maltreatment: physical abuse (PA), emotional abuse (EA), sexual abuse (SA), and physical neglect (PN). Under each type of maltreatment, four examples were given, and caregivers were asked to rate a total of 16 items with “yes (1)” or “no (0)” based on (i) case record of the child where secondary referral reasons are often included; (ii) knowledge from interacting with the participant’s biological family; and (iii) knowledge from interacting with the participant. We then recoded each type of maltreatment dichotomously according to caregiver’s ratings on the four items using the following rules: 1 (participant did experience this type of maltreatment) was given if *any* item was rated 1 by caregivers; 0 (participant did *not* experience this type of maltreatment) was given only when *all* four items were rated 0.

Information obtained from the three checklists were combined to create a final list of CAs. We first recoded the ‘other’ in the Referral Reason Checklist based on the description given by informants and derived four extra types of CAs: foster care failure, domestic violence, maternal mental illness, and parent(s) unable to cope with parenting. Where there were overlaps in the content of items across the three checklists, these were further combined into a number of dummy variables where 1 was given if one of the items was rated 1, and 0 was given when all items were rated 0. As a result, Poverty from the Referral Reason Checklist was combined with the Recipient of governmental financial aid from the Family Background Checklist into the variable ‘Poverty’; maternal mental illness in the Referral Reason Checklist and Parental mental illness in the Family Background Checklist were combined into one dummy variable called ‘Parental mental illness’; Parental imprisonment from the Referral Reason checklist and Parental criminality from the Family Background Checklist were combined into ‘parental criminality’; and finally, the four types of maltreatment variables from both Referral Reason Checklist and Maltreatment Checklist were combined into four measures of physical, emotional, sexual abuse, and physical neglect. The final list of CAs included 18 items: physical abuse, emotional abuse, sexual abuse, physical neglect, poverty, parental mental illness, unpreferable rearing condition, parental criminality, parental divorce, child’s own developmental problems, parental hospitalization, missing parents, parental death, parent(s) unable to cope with parenting, homelessness, abandonment at infancy, domestic violence, and foster care failure.

Although the checklists were based on the standard checklists developed and routinely utilized by the Japanese child protection service, these have not been previously validated in a research setting. However, we were able to obtain information about the stability and consistency of information provided over time as the project progressed. As part of a separate project, we revisited 78 participants 3 years later (2016), and re-assessed their age of first placement as well as their history of childhood maltreatment status pre-institutionalisation. Assessments were based on the Maltreatment Classification System developed by [33], in which six types of maltreatment were assessed: physical abuse, sexual abuse, emotional maltreatment, physical neglect—failure to provide, physical neglect—lack of supervision, and moral/legal/educational maltreatment. We compared physical, sexual, emotional abuse and physical neglect (combining failure to provide and lack of supervision) with the equivalent four categories in the current dataset. Cohen’s κ was run to determine the extent of agreement between the two waves with regards to the ratings of maltreatment. There was good agreement on ratings of physical abuse (κ = .66, *p* < .001), moderate agreement on sexual abuse (κ = .49, *p* < .001), and fair agreement on both physical neglect (κ = .32, *p* = .003) and emotional abuse (κ = .22, *p* = .004). Data on the age for institutionalization remained the same across the two time-frames of data collection. While slightly different measures were used across time-points and information was occasionally provided by different key caregivers, these data nonetheless provide support for consistency in ratings particularly in relation to physical and sexual abuse, as well as the presence of physical neglect.

#### Psychological and Behavioural Symptomatology

The four ‘difficulty’ subscales of the Japanese version of the Parent rating Strength and Difficulties Questionnaire (SDQ) [34] were used to assess participants’ externalising and internalizing symptomatology: hyperactivity/inattention (HI), emotional symptoms (ES), conduct problems (CP), and peer problems (PP). Caregivers rated 20 items (5 items per subscale) using a 3-point scale (0 = not true, 1 = somewhat true, 2 = certainly true) based on their observation of the target child in the most recent 6 months. Sum of the five items from each subscale formed the score for that domain of difficulty: ranging from 0 to 10, with higher scores representing more difficulty exhibited by the child in the given domain. SDQ has been reported to strongly correlate with CBCL subscales of the same constructs [34]. The internal consistency in our data (α_Total difficulties_ = .82, α_HI_ = .79; α_ES_ = .69, α_CP_ = .74, and α_PP_ = .61) are similar to those reported from a national sample of 7–15 year olds in Japan (α_Total difficulties_ = .81, α_HI_ = .76, α_ES_ = .64, α_CP_ = .54, α_PP_ = .59) [34], as well as other countries such as Finland (α_Total difficulties_ = .71, α_HI_ = .73, α_ES_ = .69, α_CP_ = .59, α_PP_ = .64) [35] and Sweden (α_Total difficulties_ = .76, α_HI_ = .75, α_ES_ = .61, α_CP_ = .54, α_PP_ = .51) [36].

### Statistical Analysis

Analyses were performed using SPSS Statistics version 23, and Mplus version 7.11 [37], following three main steps.

1. *Descriptive analysis*. We calculated the percentage of individuals who had experienced each type of CA regardless of co-occurrence. We also used a composite score of CAs (adding up all CA dummy variables) to examine the overall accumulation of CAs. We then also examined the co-occurrence of CAs, by calculating the proportion of individuals who experienced one type of CA and also experienced at least another type of CA.
2. *Identifying dimensions of CA*. Principal Component Analysis (PCA) was performed to identify underlying dimensions of CA (oblimin rotation) using SPSS Statistics Version 23. PCA was selected because it is a method that abbreviates a set of variables into fewer components that summarise their variance [38], and the primary purpose of this study is to identify and compute summary scores for the factors underlying the 18 types of CAs assessed. In determining how many principal components to retain, we conducted a parallel analysis [39] to determine the number of components that hold statistically significant eigenvalues, which represents the variance accounted for across items by the given component (sum of squared correlation coefficients between each item and the given component). Ten-thousand random datasets were generated, and 95th percentile eigenvalues were calculated for each factor. Factors from the real data with eigenvalues greater than the 95th percentile eigenvalue from the random data were retained in PCA. Component scores were then extracted.
3. *Associations with symptomatology*. Associations between the significant principal components and four SDQ difficulty subscales were estimated in a single path analytic model using Mplus 7.11, where all independent and dependent variables were assessed simultaneously, taking into account the inter-correlated nature of outcome variables. Aside from age and gender, total time spent in care was included as a covariate of no interest because all participants had experienced some degree of institutionalization, which had been identified as a significant type of adversity on outcomes. The model was adjusted for clustering to account for the nested design of the current study sample (457 participants were nested within 213 caregivers, who in turn were nested in 24 CWIs) using the clustering function in MPlus. This corrected the standard errors while retaining the same mean of each variable, which changes the t statistics, and in turn affects the p value. We also bootstrapped 10,000 times to increase confidence in results given the sample size is relatively modest for the number of parameters being estimated and clustering adjusted. Full information maximum likelihood estimation (ML) was used to include cases with missing data on the independent variables, and model fit was examined using Chi square test (non-significant p-value), Comparative Fit Index and Tucker-Lewis Index (CFI and TLI; acceptable fit = > .90), and Root mean square error of approximation (RMSEA; acceptable fit = < .08) [40].

## Results

### Descriptive Statistics

One-sample *t*-test against an age-matched Japanese normative sample [34] revealed that our institutionalized participants scored significantly higher on all four difficulty subscales of the SDQ compared to those in the general population who live with their biological parents (*p*s < .001). All SDQ subscale scores, besides Peer problems, significantly varied between males and females (HI: *r* = .164, *p* < .01; CP: *r* = .102, *p* < .05; ES: *r* = .143, *p* < .01). Age did not associate with any SDQ subscales (*p*s > .10) (Table 1), hence we removed age as a covariate from the path analysis. Total time spent in care significantly associated with HI (*r* = .139, *p* < .01) and CP (*r* = .161, *p* < .01). Since the SDQ asks informants to rate participants based on their behaviour in the past 6 months, the inclusion of participants who had been in care for less than 6 months could affect the interpretability of the findings. Therefore, we recalculated the correlation matrices after removing those participants who had been in care for less than 6 months (*n* = 25), but the pattern and effect sizes of correlations remained similar (HI: *r* = .11, *p* < .05; CP: *r* = .12, *p* < .05).

Table 3 summarizes the prevalence of the 18 CAs assessed in the current study. The most common CA was physical neglect (47.3%, *n* = 213), followed by poverty (36.4%, *n* = 163) and physical abuse (33.6%, *n* = 151). CAs highly co-occurred with one another: out of the whole sample, 80% of children experienced at least 2 or more CAs (Table 3).

**Table 3:** Co-occurrence of childhood adversities assessed

Type of CA	% (n)	% (n) of individuals who also had other type(s) of CA	Amongst those with > 1 CA, mean (SD) of number of CAs experienced
Physical neglect	47.3 (213)	97.5 (435)	2.26 (1.13)
Poverty	36.4 (163)	96.2 (429)	2.40 (1.15)
Physical abuse	33.6 (151)	96.6 (431)	2.42 (1.15)
Parental mental illness	31.0 (139)	74.4 (332)	2.36 (1.61)
Unpreferable rearing conditions	26.2 (117)	98.2 (438)	2.45 (1.22)
Emotional abuse	18.7 (84)	99.3 (443)	2.50 (1.89)
Parental crime	17.3 (78)	99.3 (443)	2.52 (1.25)
Parental divorce	13.9 (62)	98.2 (438)	2.58 (1.21)
Child’s own problem (developmental)	7.6 (34)	99.1 (442)	2.62 (1.26)
Parental hospitalisation	7.4 (33)	99.8 (445)	2.60 (1.26)
Missing parents	7.0 (31)	98.9 (441)	2.63 (1.27)
Parental death	5.6 (25)	98.7 (440)	2.65 (1.28)
Parents can’t cope with parenting	4.9 (22)	99.1 (442)	2.64 (1.27)
Homelessness	3.6 (16)	79.4 (354)	2.63 (1.27)
Sexual abuse	3.3 (15)	79.8 (356)	2.64 (1.26)
Abandonment at infancy	2.5 (11)	99.6 (444)	2.66 (1.28)
Domestic violence	.9 (4)	99.8 (445)	2.66 (1.28)
Failed foster care placement	.9 (4)	80.0 (357)	2.66 (1.28)

Number of CAs experienced	%	(n)	
1	19.7	88	
2	28.3	126	
3	29.1	130	
4	14.3	64	
5	6.1	27	
6	2.0	9	
8	.4	2	

### Underlying Dimensions of Childhood Adversity

**Fig. 1:**
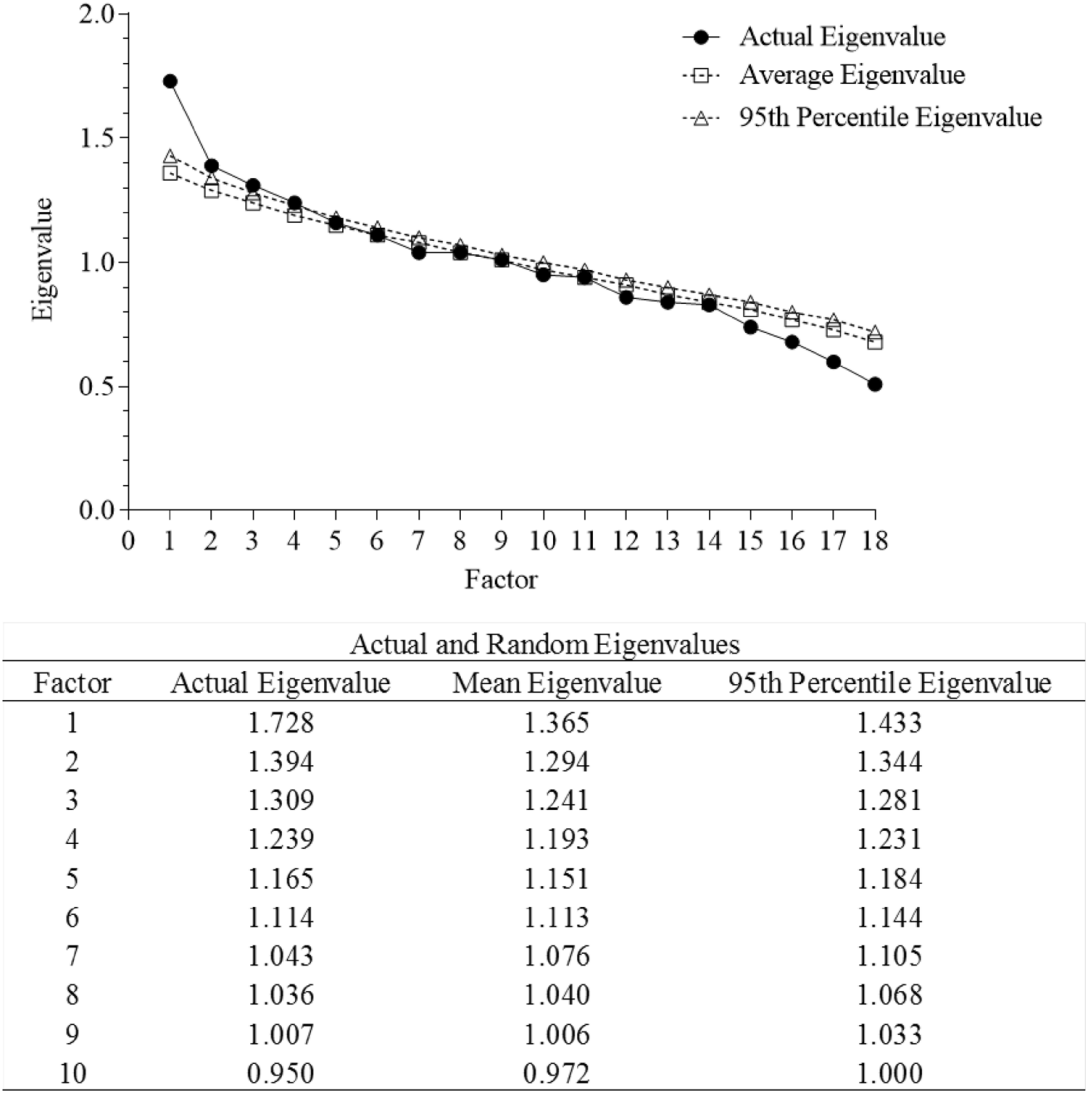
Scree plot of actual versus randomly generated eigenvalues

**Fig. 2:**
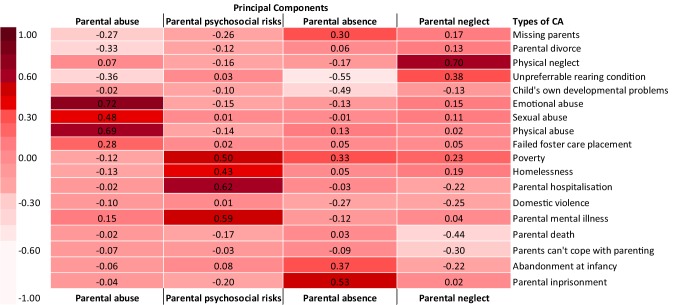
Factor loading of four principal components of childhood adversity

Parallel analysis suggested that a 4-component solution best fit the data (Fig. 1), which accounted for a cumulative variance of 31.5%. Full details of the four principal components (PC) are presented in Fig. 2. PCs were labelled based on (1) the direction of the loading between each item and the component, (2) items loading more than .30 for that particular component, and (3) the item with the strongest loading on that component. The first PC—explaining 9.6% of the variance—was related to *parental abuse*. Representative items (Item loading > .30) included physical abuse, sexual abuse, and emotional abuse. The second PC was related to *parental psychosocial risks* and explained 7.7% of the variance. Representative items included poverty, homelessness, parental hospitalization, and parental mental illness. The third PC, *parental absence*, explained 7.2% of the variance, and included primarily items of missing parents, abandonment at infancy, and parental imprisonment. The last significant PC was related to *parental neglect*, which included physical neglect and unpreferrable rearing environment, and explained 6.9% of the variance.

### Associations Between CA Components and Symptomatology

**Fig. 3:**
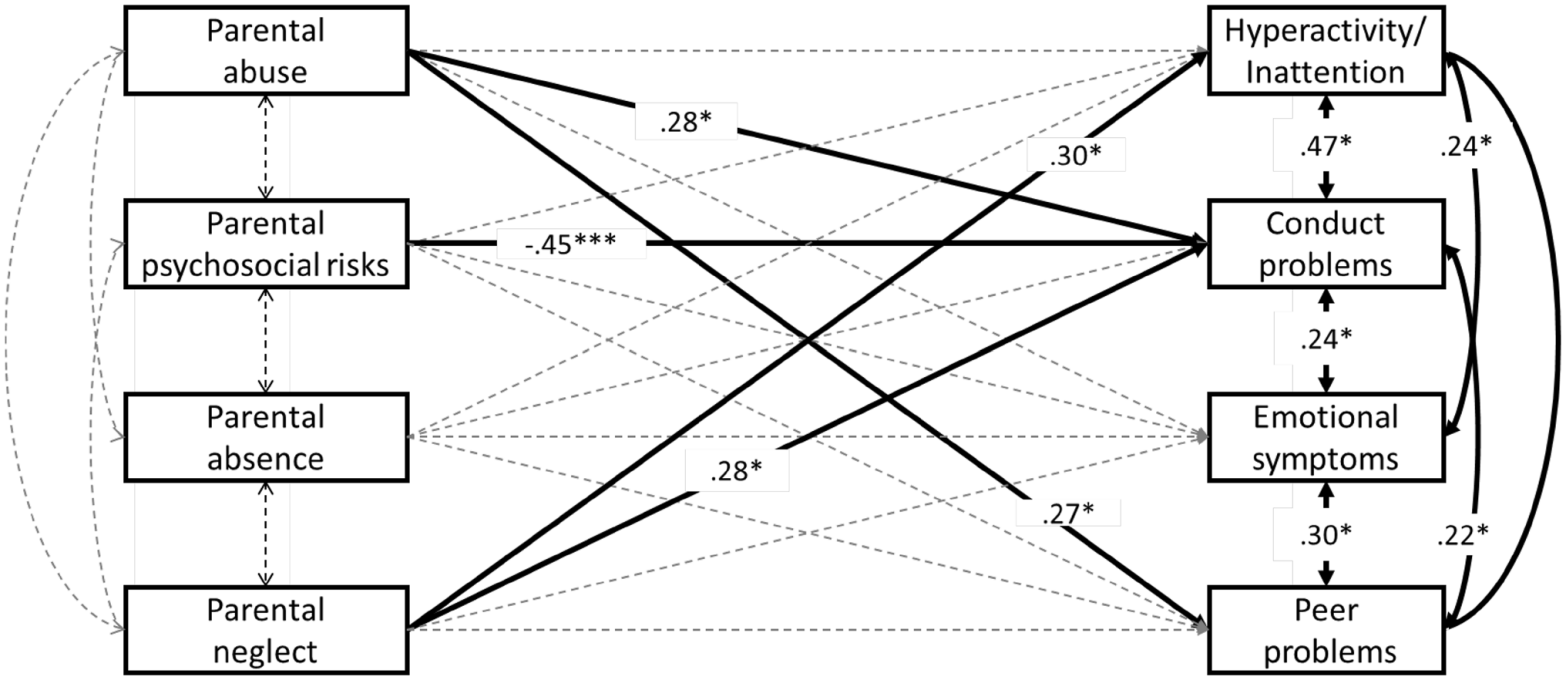
The multivariate path model showing associations between childhood adversity principal components and Strength and Difficulties Questionnaire subscales. All estimations controlled for gender and total time spent in care, adjusted for clustering at institution level, and boostrapped for 10,000 times. Full information maximum likelihood estimation (ML) was used to include cases with missing data on the independent variables. *p < .01; ** p < .05; *** p < .005. Significant associations are indicated in bold lines. p < .01; **p < .05; ***p < .005

All paths tested in the multivariate path analytic model, including associations between the four PCs and four SDQ difficulties subscales and inter-correlations between PCs and between SDQ subscales, are visualised in Fig. 3. The model fit statistics indicated good fit to the data (χ^2^(3) = .84, *p* = .84; CFI = 1.00; TLI = 1.16; RMSEA = .00, 90% CI .000–.049). Statistically significant paths are indicated as black bold lines, and non-significant paths are indicated as dotted lines in Fig. 3. Parental abuse significantly associated with CP (Std.β = .28, *p* = .03) and PP (Std.β = .27, *p* = .03). Parental neglect significantly associated with HI (Std.*β* = .30, *p* = .03), CP (Std.β = .28, *p* = .02). Parental psychosocial risks showed a significant negative association with CP (Std.β = − .45, *p* < .005). Parental absence did not show any significant association with any outcomes after clustering adjustment. Notably, ES did not associate with any PCs after the clustering adjustment (Fig. 3). Refer to Table 4 for the 95% confidence intervals of each path estimate.

**Table 4:** Path estimation standard beta and 95% confidence interval

IV	DV			
	HI	CP	ES	PP
	Std.β	Std.β	Std.β	Std.β
	95% CI [LL, UL]	95% CI [LL, UL]	95% CI [LL, UL]	95% CI [LL, UL]
Parental abuse	− .04 [− .32, .25]	.28* [.02, .54]	.17 [− .07, .44]	.27* [.04, .52]
Parental psychosocial risks	− .26 [− .51, .003]	− .45*** [− .72, − .22]	− .05 [− .25, .16]	− .17 [− .39, .05]
Parental absence	− .04 [− .32, .24]	.08 [− .18, .33]	.11 [− .11, .35]	.35 [− .18, .25]
Parental neglect	.30* [.03, .57]	.28* [.05, .51]	.16 [− .06, .39]	.21 [− .01, .43]

Model controlled for gender and total time spent in care, adjusted for clustering, and bootstrapped 10,000 times

*IV* Independent variable, *DV* dependent variable, *HI* hyperactivity/inattention, *CP* conduct problems, *ES* emotional symptoms, *PP* peer problems, *LL* lower limit, *UL* upper limit

*p < .05; **p < .01; ***p < .001

## Discussion

The aim of the current study was to investigate the underlying construct of childhood adverse experiences, and their associations with various domains of psychological and behavioural symptoms in a high-risk youth sample from Japan. Our data revealed this group of Japanese high-risk youth all experienced at least 1 type of adversity, with the majority experiencing 3 or more types of adversity. Our results indicated three key findings: (1) using principal component analysis, there were four principal components (PCs) that optimally accounted for the correlation among 18 types of adverse experiences—parental abuse, parental psychosocial risks, parental absence, and parental neglect—explaining a total of 35.1% of the variance. Each PC accounted for very similar proportions of the variance in the data (9.6%, 7.7%, 7.2%, and 6.9%); (2) Both PCs typically considered as childhood maltreatment—parental abuse and parental neglect—showed similar (conduct problems) as well as differential (parental abuse with peer problems, parental neglect with hyperactivity) associations with symptomatology; (3) parental absence did not significantly associate with any outcome.

These results partially supported the existing literature in terms of dimensionality of early experiences of adversities: two clusters emerged separately within the same category of childhood maltreatment: parental abuse versus parental neglect. In addition, parental psychosocial risks emerged as an independent component amongst the broad category of CAs. The consistency of our findings, which are based on a Japanese high-risk sample, with previous ones mostly based in community samples in Western societies suggests that the nature of adverse events and its occurrence pattern are similar across cultures, even when there are differences in prevalence rate, age of participants, or source of information. Furthermore, our results also extend previous findings of subtle differences between events comprising deprivation: distinguishing parental *absence* from parental *neglect*. The two components also held different associations with outcomes—parental neglect was associated with hyperactivity/inattention and conduct problems, whereas parental absence did not significantly associate with any outcome. Some studies have found that when impoverished living conditions are not accompanied with insecure mother–child attachment, children did not show atypical stress responses unlike children who experienced highly adverse circumstances *and* insecure attachment [41]. Others have found that after adjusting for interpersonal violence, the association between poverty and stress reactivity was diminished [6]. These findings suggest that deprivation maybe a two-pronged construct with one “prong” more closely linked with negative outcomes.

Our results from the path model also confirmed previous findings, which suggested that regardless of type and nature of the event, dimensions related to childhood maltreatment appeared to be most robustly associated with 3 out of 4 outcomes measured (i.e., hyperactivity/inattention, conduct problems, peer problems). Aside from the general effect of maltreatment, our results also suggested that parental abuse and parental neglect, when separated, hold common but also specific associations with outcomes. That is, while conduct problems was associated with both parental abuse and parental neglect, hyperactivity/inattention only significantly associated with parental neglect, and peer problems only significantly associated with parental abuse. This result further adds to previous findings where hyperactivity seems to be a behavioural consequence of severe deprivation [42], and conduct problems is more influenced by both threat and deprivation [2]. Unexpectedly, the emotional symptoms subscale did not significantly relate to any of the dimensions of adversity in our data. Several reasons can be considered. First, the emotional symptoms subscale moderately correlated with the other three SDQ subscales, and this interrelatedness was accounted for when estimating the association between the four principal components and dependent outcome variables in the path model. It is possible that externalising problems have stronger associations with adversity exposure relative to internalising problems. In fact, in the current study, emotional symptoms were reported by caregivers, not participants themselves. Indeed emotional symptoms are more elusive, and may not be as visible to external observers as externalizing problems such as conduct problems and hyperactivity. Previous studies have demonstrated weaker predictive power of informant-report compared to self-report for internalising symptoms [43]. The lower ability to accurately report internalising problems as an external observer may explain in part the absence of observed associations between PCs and emotional symptoms. Second, it is worth noting that emotional symptoms did significantly associate with parental abuse (*r* = .13, *p* < .01) and parental neglect (*r* = .10, *p* < .05) in a simple two-tailed correlation analysis, and boys and girls showed similar correlation patterns (Supplemental Table 1). However after we adjusted for covariates and clustering, a relatively conservative approach, this association no longer remained significant.

Finally, the parental psychosocial risk component supported previous findings that events reflect parental malfunctioning [4] and maladaptive family functioning [5] tend to cluster together. However, unexpectedly, in our data, this PC negatively associated with conduct problems. To follow up on this unexpected negative association, we ran a simple correlation between the PC and Conduct Problem subscale (*r* = − .16, *p* < .01), as well as for each subscale item (*r* = − .11 ~ − .19, *p*s < .05) (Supplementary Table 1). It may be that with the effects of maltreatment (i.e., abuse, neglect) removed by the two PCs of parental abuse and parental neglect, parental psychosocial risks not accompanied with violence against children, is no longer harmful within this high-risk sample. It is noteworthy that a negative association does not necessarily mean that parental psychosocial risks are protective against negative outcomes, given that this association is derived from an extremely high-risk sample. Thus, this same negative association may not generalise to the population, where the frequency and severity of adversities differs.

While the current study findings have exciting implications both theoretically and clinically, there are several limitations that should be noted. The first set of limitations relate to how CAs were assessed retrospectively. Although the use of self-reports, informant (e.g., parent) reports, and/or case records to code for and rate CAs are considered the gold-standard methods for assessing early-life adversity, these methods nonetheless have the potential for bias and under-reporting. Furthermore, in the case of retrospective self-reports, these could yield recall bias resulting in over- or under-reported events. For example, a longitudinal investigation of childhood maltreatment [44] demonstrated that self-reports of sexual and physical abuse are highly unstable over time. In the current study, due to ethical and safeguarding restrictions, we did not have permission to ask participants themselves about their previous experiences of the CAs, nor could we access their case records directly. Therefore, relying on the participant’s key caregivers to provide information for the assessment of CAs using a standardized official checklist and two project-developed checklists was the only viable option. Although reports from child care professionals are more likely to rely on judgments based on objective events (via case records) relative to self- or parental-report of adverse experiences, similar to case records, there may also be a tendency to under-report adverse experiences due to unawareness. Moreover, even though some information may be elicited through daily interactions with children and young people, some participants may be less communicative than others. Although there are concerns about the way we rated the CAs, we applied dichotomous ratings for each item in the checklist since our objective was to assess the overlap between different types of CAs. Moreover, it was essential to obtain information as accurate and consistent as possible across caregivers while minimising task demands on them. Information such as CA’s age of onset, duration, and intensity were not included in the current study due to challenges of getting this information from case records in a consistent manner across participants. Future studies assessing early adverse experiences should ideally involve multiple sources and informants [45], and use more refined measures of a broad range of early adverse experiences, as well as include age of onset, or length of exposure to adversities.

Another limitation of the study is the range of CAs that were explored. Although, we determined the items through multiple participatory meetings by institution caregivers, social workers, and institution directors, and used the option of ‘other’ to prompt any adverse events not included in the checklists, there may still be events that were excluded, such as exposure to peer victimization, which was difficult to assess based on case records. We also did not measure adversity associated with institutional care, or the effects of institutionalization per se, given that all participants had experienced this. In addition, we also had not considered participants’ adaptation to institutional life, which has been consistently identified as a risk factor for developmental outcomes such as internalizing and externalising outcomes. Instead, we controlled for total time spent in care in the path analysis, and correlated children’s total time in care with four SDQ difficulties subscales. Interestingly, we found that the longer children were in care, the more elevated the level of externalising problems, namely hyperactivity/inattention and conduct problems. This finding supports the association previously reported by other researchers between deprivation and externalising problems. This result remained significant even after we removed children who had been in the current institutional care for less than 6 months. Although it is not common for children to leave residential care in Japan once placed—for example in 2015 6.9% (*N* = 2735) of institutionalised children had either gone back home (*n* = 2597; 6.6%), were adopted (*n* = 24; .06%), or moved to foster homes (*n* = 114; .2%) [46]—it is important to note that this association does not infer directionality. It is possible that children with higher levels of externalising problems would be more likely to remain in residential care for longer compared to those with milder or no such symptoms. Without longitudinal data, it is difficult to disentangle these possibilities.

Finally, we only used caregiver reports on participants’ internalising and externalising outcomes as nearly 40% of the participants were under the age of 11 years old, for which a self-report version of the SDQ is unavailable. As such, for consistency, we relied on caregiver reports for all participants. However, previous data has found that parental reports of symptomatology, compared to self-reports, showed weaker associations with some symptom outcomes, especially with internalizing problems, which may be more difficult to detect by external raters [43]. Another potential issue is same-rater biases given that the same individual rated both the adversity and the symptom outcomes. Future research in this area, while promoting measurement development in different cultural/language settings, should also aim to collect multi-rated data to ensure maximal validity of the construct. Moreover, in the current study, the institutionalized youth are closely supervised by institutional caregivers, hence, certain behavioural indices (e.g., suicide and self-harm, risky sexual behaviour) are relatively rare, and were not included in outcome assessments, despite their known association with early adversity. Furthermore, ethical and safeguarding issues prevented us from asking young people about suicidal ideation. Future research should consider including a broader range of outcome measures.

The current study, aimed to examine temporal associations between ‘distal’ adverse events in childhood with later on problem-behaviours, demonstrated that early adverse experience is a multi-dimensional phenomenon. While different types of maltreatment holds unique associations with outcomes, maltreatment as a whole is the most robust predictor for psychological and behavioural symptoms. This emphasises the importance for practitioners in social services and policymakers to take concrete steps towards establishing and enforcing laws for the prevention, as well as intervention, for childhood maltreatment. Furthermore, since the two forms of deprivation, when distinguished, have fundamental differences in their impact on children’s developmental outcomes, it is extremely useful for intervention studies to reduce the negative effect of poverty, community violence, or other forms of adversity, by promoting a healthy relationship between the children and their caregiver. Moreover, for Japan specifically, where the child protection system is currently undergoing reform, service providers should emphasise the importance of assessing adverse experiences of children prior to protection, and use the information to guide the development of more effective individualized care plans, especially if these results hold are replicated. Future studies using longitudinal prospective design will be more robust and informative for drawing causal relationships between these dimensions of environmental input and outcomes. Follow-up studies on the same group of young people on how the effects of different dimensions of adverse experiences persist will also be useful for understanding the long-term consequences, and shed light in the role of culture in response to adversity, which has been over looked [47].

## Summary

In the current study, we assessed the latent structure of 18 types of childhood adversity experienced by 457 high risk Japanese children and adolescents. Four distinct dimensions emerged: parental abuse, parental neglect, parental psychosocial risks, and parental absence. A path analytic model revealed both shared and specific association between the principal components and symptomatology: parental abuse and parental neglect together significantly associated with elevated conduct problems; while parental neglect specifically contributed to heightened hyperactivity/inattention, parental abuse uniquely associated with peer problems.

## Electronic supplementary material

Below is the link to the electronic supplementary material.

Supplementary material 1 (DOCX 22 KB)
