# Supplementary material for: Dimensionality of Early Adversity and Associated Behavioral and Emotional Symptoms: Data from a Sample of Japanese Institutionalized Children and Adolescents
Source: Child Psychiatry Hum Dev. 2018 Oct 27;50(3):425–38. doi: 10.1007/s10578-018-0850-4 (PMC6478638; doi:10.1007/s10578-018-0850-4)
Supplement: Supplementary file 1 — Supplementary material 1 (DOCX 22 KB) [file 10578_2018_850_MOESM1_ESM.docx]

# Supplementary Table 1. Spearman’s correlation coefficients between SDQ difficulties subscales, items of Conduct problems, and Principal components.

|  | Principal components | | | | | | | | | | | | | |
| --- | --- | --- | --- | --- | --- | --- | --- | --- | --- | --- | --- | --- | --- | --- |
|  | All | | | |  | Male | | | |  | Female | | | |
| Strength and difficulties Questionnaire | PC1 | PC2 | PC3 | PC4 |  | PC1 | PC2 | PC3 | PC4 |  | PC1 | PC2 | PC3 | PC4 |
| - Hyperactivity/inattention | .02 | -.09 | .01 | .10* |  | .04 | -.10 | .03 | .06 |  | .03 | -.09 | .00 | .15* |
| - Emotional symptoms | .13** | -.01 | .04 | .10* |  | .13* | -.01 | .03 | .05 |  | .11 | -.01 | .04 | .16* |
| - Peer problems | .12* | -.09 | .02 | .07 |  | .19** | -.02 | .01 | .03 |  | .05 | -.17* | .03 | .12 |
| - Conduct problems | .07 | -.16** | .04 | .09 |  | .06 | -.14* | .10 | .09 |  | .10 | -.20** | -.04 | .09 |
| *-Item 5* |  | -.11* |  |  |  |  | -.07 |  |  |  |  | -.18* |  |  |
| *-Item 7* |  | -.19** |  |  |  |  | -.18** |  |  |  |  | -.22** |  |  |
| *-Item 12* |  | -.12* |  |  |  |  | -.10 |  |  |  |  | -.16* |  |  |
| *-Item 18* |  | -.18** |  |  |  |  | -.15* |  |  |  |  | -.22** |  |  |
| *-Item 22* |  | -.13** |  |  |  |  | -.14* |  |  |  |  | -.13 |  |  |
| PC1: Parental abuse; PC2: Parental psychosocial risks; PC3: Parental absence; PC4: Parental neglect; Item 1: Often has temper tantrums or hot tempers; Item 7: Generally obedient; Item 12: Often fights with other children; Item 18: Often lies or cheats; Item 22: Steals from home, school or elsewhere; * p < 0.05; ** p < 0.01; | | | | | | | | | | | | | | |
